# DNA barcoding as a tool for robust identification of cervids of India and its utility in wildlife forensics

**DOI:** 10.1080/23802359.2018.1438858

**Published:** 2018-02-20

**Authors:** Ved P. Kumar, Malay Shukla, Ankita Rajpoot, Mukesh Thakur, Parag Nigam, Dhyanendra Kumar, Anil Kumar Mehta, Surendra P. Goyal

**Affiliations:** a Wildlife Forensic Cell, Wildlife Institute of India, Dehradun, Uttarakhand, India;; b Department of Zoology, Veer Kunwar Singh University, Arrah, Bihar, India;; c Institute of Forensic Science, Gujarat Forensic Sciences University, Gandhinagar, Gujarat, India;; d Molecular Systematics Laboratory, Zoological Survey of India, Northern Regional Centre, Dehradun, Uttarakhand, India;; e Molecular Systematics Division,Centre for DNA Taxonomy, Zoological Survey of India, Kolkata, India

**Keywords:** DNA barcoding, cytochrome c oxidase subunit I (COI), intraspecies and interspecies, wildlife forensic and conservation biology

## Abstract

DNA barcoding has become a popular method of choice for identification of specimen based on molecular techniques. Here, we present preliminary findings on generating robust DNA barcode library of Cervids of India. The dataset comprising the DNA barcode library of seven deer species included in the genus *Cervus, Axis* and *Muntiacus* classified under family Cervidae. Mitochondrial Cytochrome C Oxidase subunit I gene of ca. 710 bp accepted widely as DNA barcode region, was used for generating species specific signature from 31 known samples of seven Indian deer species. Expectedly, the NJ tree clustered three genera i.e. *Cervus, Axis* and *Muntiacus* of Cervids of India into three clades. Further, the intra- and interspecies distances based on Kimura 2 parameter model also supported the results. The average intra- and interspecies sequence divergence were 0.011 (±0.09) and 0.65 (±0.14), respectively. The present study has exhibited that DNA barcoding has discriminating power to delineate boundaries among the closely related species. The data generated are of high importance to the law enforcement agencies in effective identification of species in wildlife offence cases. The similar approach can be utilized for generating DNA barcodes for other Indian mammals for making effective management and conservation action decisions.

## Introduction

Indian subcontinent is a home to four mega biodiversity hot-spots among the 35 hot-spots around the world as defined by Conservation International (Myers et al. 2000). Of the known species that inhabits the Indian sub-continent, few species are endemic while many species have flourished through the course of evolution.

Majority of deer species are classified under family Cervidae, which is the second most diverse group in the world after family Bovidae. Deer are among the most abundant, visible and wondrous mammal with around 40 existing species in the world (Gaur et al. 2003). These deer (Cervidae) species are classified into sixteen genera and two subfamilies with five tribes (Gilbert et al. 2006; Hassanin et al. 2012). The subfamily, Cervinae composed of two tribes *Cervini* (with four genera i.e. *Cervus, Axis, Dama*, and *Rucervus*), the second is *Muntiacus* (genus: *Muntiacus* and *Elaphodus*) (Gilbert et al. 2006). However, in Indian subcontinent, seven deer species are present and classified under three genera namely *Cervus*, *Axis* and *Muntiacus*, where genus *Cervus* consisted with four deer species i.e. Hangul (*Cervus hanglu hanglu*), Swamp deer *(Rucervus duvaucelii*), Sambar (*Rusa unicolor*) and Brow antlered deer (*Rucervus eldii*); genus *Axis* composed with a species i.e. Chital (*Axis axis*) along with a sub-species Hog deer (*Axis porcinus*) and lastly, genus *Muntiacus* is composed of single species i.e. Barking deer (*Muntiacus muntjak*). In India, deer species are protected under the Wildlife (Protection) Act, 1972 (WPA) under different schedules (I, II, III and IV), moreover all species also protected under International Union for Conservation of Nature (IUCN) and Convention on International Trade in Endangered species (CITES) in different categories and appendix, respectively (IUCN, 2016; CITES, 2016). The detail conservation and population status are given in Table 1.

**Table 1.** List of seven Indian deer species, the number of individuals, Genus, distribution and conservation status of each used in the present study.

					Distribution in India	Status		
S. no	Species name	Genus	Common name	No. of individuals	Population trend	IUCN 2017	CITES 2015	WPA
1	*Rusa unicolor*	*Cervus*	Sambar	5	Throughout India	Vulnerable A2cd +3cd +4cd ver. 3.1	Appendix-II	Schedule-II
					Decreases			
2	*Rucervus duvaucelii*	*Cervus*	Swamp deer	5	Fragmented population present in Central, Northern-eastern Indian	Vulnerable C1. Ver. 3.1	Appendix-I	Schedule-I
					Decreases			
3	*Cervus hanglu hanglu*	*Cervus*	Hangul	5	Single population in Dachgram national park J&K, India	Critically Endangered	Appendix -I	Schedule-1
					increasing			
4	*Rucervus eldii*	*Cervus*	Brow antlered deer	3	Single population in Manipur	Endangered A2cd +3cd +4cd Ver. 3.1	Appendix -1	Schedule-I
					Decreases			
5	*Axis axis*	*Axis*	Chital	5	Throughout India	Least concern ver. 3.1	Appendix -II	Schedule II
					Unknown			
6	*Axis porcinus*	*Axis*	Hog deer	3	Fragmented population present in northern-western India	Endangered A2bcd Ver. 3.1	Appendix -I	Schedule-I
					Decreases			
7	*Muntiacus muntjak*	*Muntiacus*	Barking deer	5	Throughout India	Least Concern Ver. 3.1	Appendix -II	Schedule-II
					Decreases			

IUCN:  International Union for Conservation of Nature; CITES: the Convention on International Trade in Endangered Species of Wild Fauna and Flora; WPA: Wildlife Protection Act.

Last few decades, saw an unprecedented onslaught on the wild fauna as a result of poaching and habitat fragmentation. Deer are generally considered as a prey species to the large carnivores and forms an integral part of an ecosystem, hunted primarily for local consumption as well as for commercial benefits (Qureshi et al. 2004; Tordoff et al. 2005; Maxwell et al. 2007; Johnson 2010). Reckless poaching of deer has resulted in mortification of fragile ecosystem since the prey density becomes low, ultimately effecting food availability for carnivores like tigers, leopards, etc. At times during, wildlife crime, meat samples seized are in horrible condition and sometimes samples of multiple species are mixed in the single container and send for forensic examination. In such cases, it is necessary to generate a unified system for analysis of wildlife samples that can substantially delineate the species identification.

For over a long time, molecular genetics has been an indispensible tool for species identification, taxonomic classification and their correlation (Parson et al. 2000; Budowle et al. 2003; Ortea et al. 2009; Laakmann et al. 2013; Rajpoot et al. 2017). DNA barcoding is a simple technique with complex applications in biodiversity analysis and its assessment to generate a digital identification system. The cytochrome c oxidase subunit 1 (COI) has been designated as DNA barcode loci for mtDNA-based identification of animal specimens (Folmer et al. 1994; Hebert, et al. 2003). Few last decades have resulted in an exponential rise in the popularity of DNA barcoding to address different biological questions related to biodiversity using complex biological samples (Hebert, et al. 2003; Lahaye et al. 2008; Hajibabaei et al. 2011). The DNA barcoding methodology depends on the assumption that each species will have similar DNA barcodes representing its intraspecies variability. Information based on DNA sequences have widely been used in systematic, phylogenetic, phylogeography and species identification in wildlife forensics to address different biological questions (Hebert et al. 2004). The present study addresses the need for comprehensive DNA barcode database of all seven deer species in India to better integrate the wildlife management and wildlife law enforcement in India.

## Materials and methods

### Sample collection and laboratory procedure

A total of 31 samples of seven deer species inhabiting India were collected from different regions and stored as dried/fresh tissue in cryo vials till processing them for DNA extraction. Seven currently surviving Indian deer species i.e. Sambar (*n* = 5), Hangul (*n* = 5), Swamp deer (*n* = 5), Chital (*n* = 5), Hog deer (*n* = 3), Barking deer (*n* = 5) and Brow antlered deer (*n* = 3) were used in this study (Table 1).

Genomic DNA was isolated from these samples using the Qiagen DNeasy Blood and Tissue Kit (Qiagen, Valencia, CA) according to the instruction manual. Extracted DNA templates were amplified by polymerase chain reaction (PCR) using the universal primer of COI (Folmer et al. 1994).

The amplification through PCR (ABI 2700 Thermo Cycler) was executed using 2× PCR master mix (Thermo Fisher Scientific, Waltham, MA), 4 pm of each primer and approximately 45–50 ng of genomic DNA under subsequent condition: initial denaturation at 95 °C for 5 min, followed by 40 cycles of denaturation at 94 °C for 35 s, primers annealing at 45 °C for 1 min, extension at 72 °C for 45 s with a final extension at 72 °C for 15 min. Thereafter, 3.5 µl of PCR products were subjected to electrophoresis on 1.5% agarose gel and visualized over the transilluminator to detect the amplification. During the whole procedure, extraction and PCR blanks were incorporated into the analysis to check the contamination. Bidirectional sequencing of COI gene was performed using Big dye terminator cycle sequencing kit® v 3.1 (Kumar et al. 2014). The sequences reported in this paper have been submitted in NCBI GenBank and accession numbers are awaited.

### Data analysis

Sequence data generated were analyzed by Sequence Analysis software v 5.2 (Applied Biosystems, Foster City, CA) and the forward and reverse sequences were trimmed and assembled using Cromas 2.6.4 (http://www.technelysium.com.au). After validating all obtained sequence data, multiple sequence alignment (MSA) was performed using CLUSTAL W as implemented in BioEdit v 7.0.9.0 software (Hall 1999). The intraspecies and interspecies distances were calculated using Kimura 2 parameter (K2P) model (Kimura 1980) as recommended by the Consortium for Barcode of Life (CBOL, http://www.barcoding.si.edu/protocols.html) using MEGA 7.0 software (Kumar et al. 2016). Evolutionary distances were calculated using neighbor-joining (NJ) method (Saitou and Nei 1987), and the phylogenetic tree was constructed with 1000 bootstrap replicates (Felsenstein 1985) using MEGA 7.0 (Kumar et al. 2016). The species identification success rate was calculated using genetic distance and BLAST methods. Reference DNA sequence of musk deer was downloaded from GenBank and was used as out-group for the analysis.

## Results and discussion

### Divergence assessment

The isolated genomic DNA for all 31 samples was of good quality. The obtained novel sequences of COI (658 bp) contained 479 conserved regions, 129 variable sites, 71 parsimony sites and 58 singleton sites. The BLAST result of COI sequences indicated that all seven Indian deer samples were matched with 99–100% with respective species. The maximum likelihood estimate of transition/transversion bias (R) was 11.18 whereas nucleotide composition of COI loci was A = 31.8%; T = 24.6%; C = 27.1% and G = 16.6%. Furthermore, the average intraspecies mean pairwise difference was 0.11 (± 0.09), while average interspecies sequences divergence was 0.65 (± 0.14). The observed variable sites position within the COI sequences of seven Indian deer species are shown in Table 2.

**Table 2.** Variable sites position in COI mitochondrial loci among Indian Cervids.

																														

### Determination of intraspecies and interspecies K2P distances

**Figure 1.**
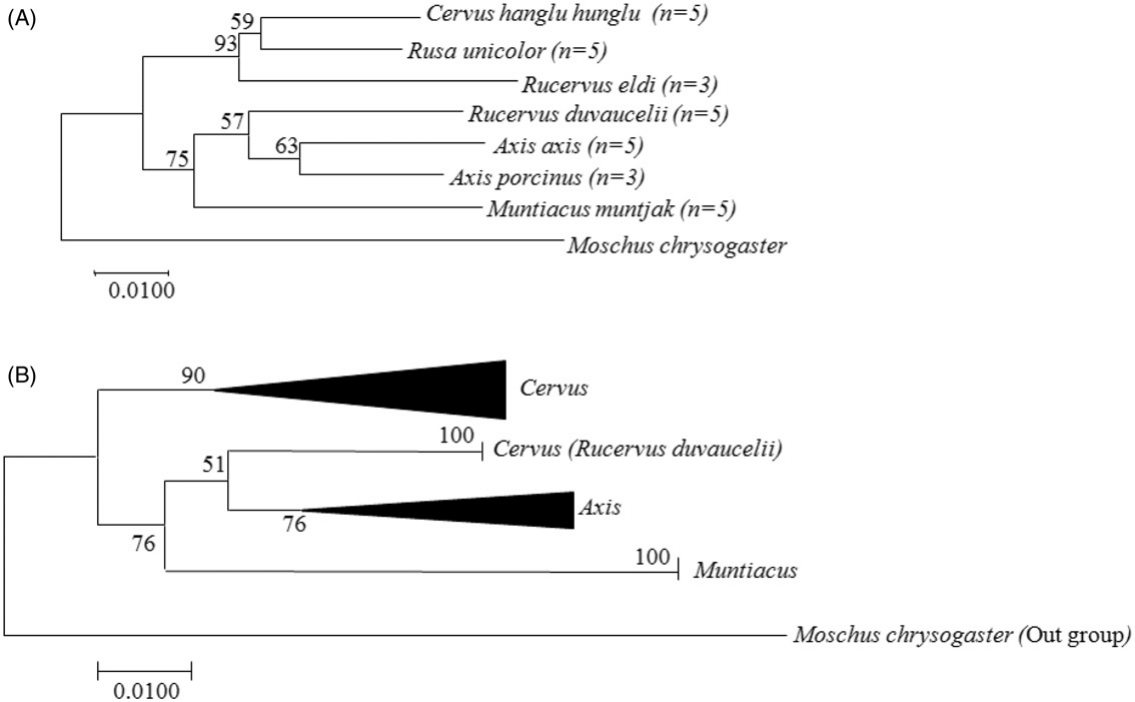
The evolutionary relationship among Indian Cervidae using the Neighbor-Joining method (NJ) undertaken in MEGA 7.0. (A) Topology showing the intraspecies (species level) relationship between seven Indian deer species and (B) topology showing interspecies (genus level) relationship.

The obtained COI sequences clearly showed intraspecies and interspecies distance among seven deer species. The tree topology revealed that these seven Indian deer species were divided into two major groups, where clear intraspecies (within species level) and interspecies (genus level) distances were observed by using a K2P technique (Figure 1). Among four species from genus *Cervus* included in the study, NJ tree, clustered an array of three species in one group (Hangul, Sambar and Brow antlered deer) with node support 59–93%, while Swamp deer was clustered in another group with genus *Axis*. The second group formed by genus *Axis* (Chital and Hog deer) and *Muntiacus* (Barking deer), also had an interesting inclusion of Swamp deer together with genus *Axis* as sister group with 57% node support. In genus *Axis*, the intraspecies K2P distance based on tree topology showed that Chital and Hog deer are clustered together with 63% node distance. Whereas in genus *Muntiacus*, Barking deer clustered separately from genus *Axis* with node distance of 75%. The interspecies distance in NJ tree also divided these three genera clearly with tree ranged from 52% to 91%.

**Figure 2.**
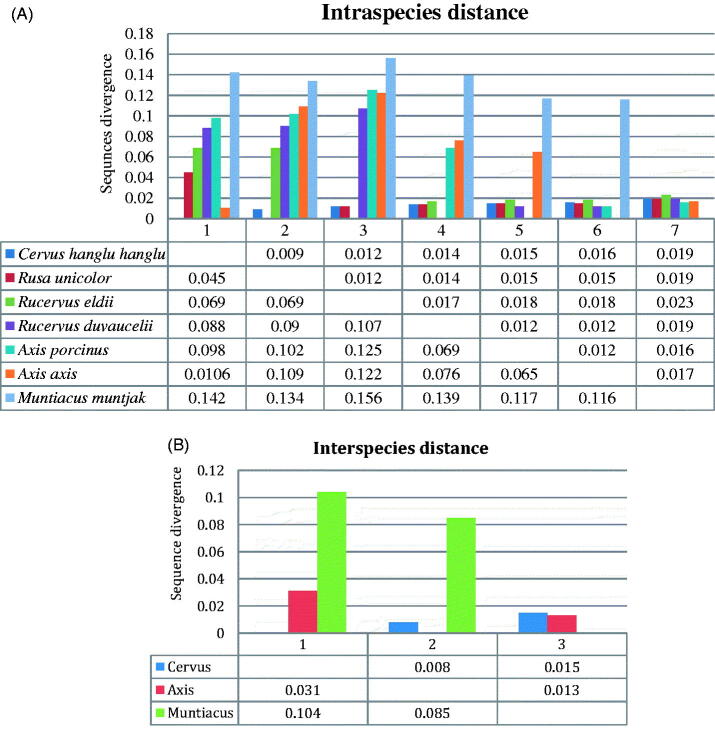
Estimates of evolutionary divergence over sequence pairs intraspecies (A) and interspecies (B). The numbers of base differences per site from averaging over all sequence pairs between groups are shown for *n* = 31 nucleotide sequences after removing all ambiguous positions for sequence pairs.

The evolutionary divergence based on intraspecies variation, in seven deer species, ranged from 0.45 (± 0.09) to 0.156 (± 0.023), whereas the maximum sequences divergence (0.156; ± 0.023) were observed between Brow antlered deer and Barking deer (Figure 2(A)). Between *Cervus* group the sequences divergences ranged from 0.045 (± 0.09) to 0.117 (± 0.016), where the maximum divergence (0.117; ± 0.016) was observed between Brow antlered deer and Swamp deer, whereas minimum divergence was observed (0.045; ± 0.09) between Hangul and Sambar. Moreover, in the *Axis* group, observed sequence divergence from Chital to Hog deer was 0.065 (± 0.012), while in *Muntiacus*, sequence divergence was absent since Barking deer was solitary representative of the group, albeit it showed minimum sequence divergences (0.116; ±0.017) from Chital (Figure 2(A)).

The evolutionary divergence based on interspecies variation, in three genus ranged from 0.031 (± 0.008) to 0.104 (± 0.015), among which, the maximum (0.104; ± 0.015) and minimum (0.031; ± 0.008) sequences divergence was observed between the genus *Cervus* to *Muntiacus* and *Cervus* to *Axis,* respectively. Moreover, the sequence divergence between *Axis* to *Muntiacus* was 0.085 (± 0.013) (Figure 2(B)). The DNA sequences of COI gene revealed that the obtained sequences are very helpful to delineate the Indian Cervids.

## Conclusion

The present study unequivocally demonstrates the applicability of DNA barcodes using COI gene as a potential tool for identification of Cervids in Indian. Our data represent the validity of DNA barcodes in identification of mammalian species especially closely related deer species and a paradigm shift to focus on conservation of mammals through robust tools like DNA barcodes. These barcodes once developed could become potent tools in the hands of enforcement agencies entrusted with the responsibility of checking their illicit trade. In cases where meat samples, raw or finished products of deer species are seized, approach based on the present study would help in robust identification of closely related/multiple species Universally accepted DNA barcode gene when used in concatenation with other mitochondrial gene can deliver information on phylogeographic perspective of the data that would enable law enforcement agencies to track the geographic origin of the wild specimens and their derivatives in illicit trade. An effective check on their collection from wild, in turn, would help in their conservation in situ and wildlife management.
